# Localized Surface Plasmon Resonance Sensing and its Interplay with Fluidics

**DOI:** 10.1021/acs.langmuir.4c00374

**Published:** 2024-04-29

**Authors:** Nikhil Bhalla, Amy Q. Shen

**Affiliations:** † Nanotechnology and Integrated Bioengineering Centre (NIBEC), School of Engineering, Ulster University, Belfast BT15 1AP, United Kingdom; ‡ Healthcare Technology Hub, School of Engineering, Ulster University, Belfast BT15 1AP, United Kingdom; § Micro/Bio/Nanofluidics Unit, Okinawa Institute of Science and Technology Graduate Univerisity, Onna-son, Okinawa 904-0495, Japan

## Abstract

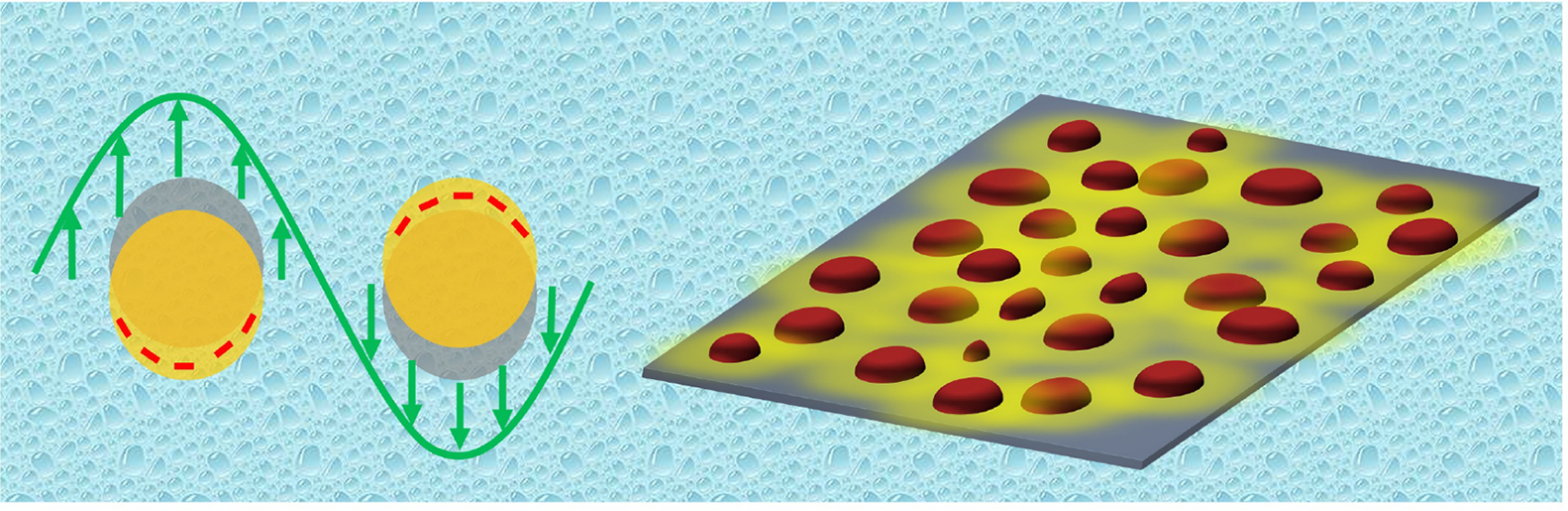

In this Feature Article, we discuss the interplay between fluidics and the localized surface plasmon resonance (LSPR) sensing technique, primarily focusing on its applications in the realm of bio/chemical sensing within fluidic environments. Commencing with a foundational overview of LSPR principles from a sensing perspective, we subsequently showcase the development of a streamlined LSPR chip integrated with microfluidic structures. This integration opens the doors to advanced experiments involving fluid dynamics, greatly expanding the scope of LSPR-based research. Our discussions then turn to the practical implementation of LSPR and microfluidics in real-time biosensing, with a specific emphasis on monitoring DNA polymerase activity. Additionally, we illustrate the direct sensing of biological fluids, exemplified by the analysis of urine, while also shedding light on a unique particle assembly process that occurs on LSPR chips. We not only discuss the significance of LSPR sensing but also explore its potential to investigate a plethora of phenomena at liquid–liquid and solid–liquid interfaces. This is particularly noteworthy, as existing transduction methods and sensors fall short in fully comprehending these interfacial phenomena. Concluding our discussion, we present a futuristic perspective that provides insights into potential opportunities emerging at the intersection of fluidics and LSPR sensing.

## Introduction

The discovery of localized surface plasmon resonance (LSPR) has laid the cornerstone for multifarious research domains, spanning from material science to biosensing, as documented in prior works.^1,2^ LSPR is considered as a transduction platform, holding the promise of facilitating the development of cost-effective and portable devices for a wide array of applications, as highlighted by recent studies.^3,4^ LSPR, fundamentally an optical phenomenon, manifests as a synchronized oscillation of valence electrons within noble metals, leading to the absorption of light within the ultraviolet–visible (UV–vis) spectrum. This phenomenon arises from interactions between incident photons and the conduction band of a noble metal nanostructure. An exponentially decaying length of the electromagnetic field is also observed in LSPR, which is on the order of 5–10 nm.^1,5^ This short decay length in the LSPR is sensitive to the interference caused by fluctuations in the refractive index of the solution in close proximity to the surface (5–10 nm). This unique feature forms the basis for applications in refractive-index-based LSPR bio/chemical sensing, opening avenues for innovative research and technological advancements in the biosensing field.

From classical physics perspective, LSPR can be modeled by Mie’s solution to Maxwell’s equation, yielding an empirical expression for extinction, *E*(λ), which is dependent on the dimension, shape, density, and local environment of the nanostructure:^5^
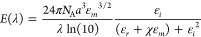
1In eq 1, *N*_A_ represents the areal density of the nanostructure, *a* denotes the average radius of the nanostructure (modeled as a sphere), λ is the wavelength of the absorbing radiation, ε_*m*_ stands for the dielectric constant of the medium enveloping the nanostructure (assumed to be a positive, real integer that is independent of λ), ε_*i*_ signifies the imaginary component of the nanostructure’s dielectric function, ε_*r*_ denotes the real component of the nanostructure’s dielectric function, and χ is the term employed to characterize the aspect ratio of the nanostructure. Note that the value of *a* should be less than λ (absorbed by the nanostructure).

The influence of the local change in the refractive index around the nanostructure on λ can be assessed through absorption spectroscopy, such as by employing simple ultraviolet–visible–infrared (UV–vis–NIR) spectroscopy methods, particularly if the nanostructure absorbs light within the UV–vis–NIR wavelength range. The changes in the wavelength can be described by
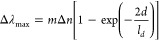
2where *m* is the bulk refractive index response of the nanostructure, *d* is the effective adsorbate layer thickness and *l*_*d*_ is the characteristic electromagnetic field decay length. It should be noted that the refractive index *n* is a function of dielectric constant, ε, *n* = ε^1/2^. Equation 2 shows that changes in the peak wavelength Δλ are directly proportional to Δ*n*, i.e., the local change in the refractive index around the nanostructure. This concept, particularly highlighted by eq 2, serves as the cornerstone of LSPR biosensing, wherein variations in wavelength are directly linked to the binding of biomolecules and analytes (such as cells, proteins, DNA, etc.) onto the nanostructure’s surface.^14^ Different nanoarchitectures have been developed to enhance the sensitivity of the LSPR biosensors, i.e., to maximize the Δλ upon interaction of biomolecules with nanostructures. These variations encompass a wide array of nanostructures, including spherical nanoparticles, intricate shapes like cubes and stars, core–shell structures, and even pillared mushroom-like configurations.^6,15−18^ Additionally, these architectures employ an extensive range of materials, including gold, silver, graphene, bimetallic structures, multimetallic compositions, and emerging 2-D materials like MXenes.^19−24^Table 1 also shares some of these LSPR substrates, which are combined with microfluidics to form bioassay chips.

**Table 1:** Different Nanoarchitectures Developed for LSPR-Based Microfluidics Bioassay Systems

type of LSPR substrate	sensing features	area of broad application	ref
nanospikes	comprising gold with light absorption at 513 nm; limit of detection of 0.5 pM for SARS-CoV-2 spike protein in diluted human plasma; sensitivity of 183 nm/RIU	protein immunoassays	(6)
nanoislands	comprising gold; spherical sphere-like islands of 40 nm; trapping the dielectric polystyrene beads, low-density lipoproteins and exosomes	*in situ* nano-objects trapping and detection	(7)
nanomushrooms	comprising silicon dioxide and gold; light absorbance between 530 and 580 nm depending on the size of gold cap on the silicon dioxide stem; limit of detection in range of zeptomolar achieved for protein detection; sensitivity between 80 and 90 nm/RIU	used for cell study, DNA and protein-based bioassays	(8−11)
nanostars	dual-mode sensing in combination with surface-enhanced Raman spectroscopy; plasmonic coupling of substrate and nanostars inside microfluidic setup	protein immunoassays	(12)
nanotriangles	comprising silver; height of 50 nm and width of 100 nm	protein bioassays	(13)
nanoellipsoids	made up of anodic aluminum oxide and gold; sensitivity of 240.9 nm/RIU	protein immunoassays	(7)

In our prior research endeavors, we concentrated on advancing the development of gold, silver, and bimetallic nanostructures.^8,25−27^ These efforts culminated in the creation of extensive nanostructured surfaces, capable of accommodating various biomolecules, ranging from prokaryotic and eukaryotic cells to amino acid- and nucleic acid-based biomolecules.^8,9^ An essential aspect of our research has been the exploration of the nanomushroom (NM) architecture,^10^ which has yielded significant results, particularly in establishing a platform for long-term cell monitoring.^8^ Previously, nanostructures were known to induce toxic effects.^28^ However, our unique mushroom-like nanostructures, resembling villi-like structures in the body preferred by cells, have played a pivotal role in ensuring high biocompatibility of LSPR sensors.^29−31^ Consequently, our developed LSPR platforms, especially NM-based substrates, provide a more conducive environment for cell proliferation, overcoming the previous toxicity challenges. Furthermore, we integrated the NM architecture with microfluidics to achieve high sensitivity in single nucleic acid studies.^32^ We have also developed portable systems and integrated LSPR with semiconductor technology, facilitating the transition of LSPR technology from academia to industry.^33,34^

## Creating an Integrated LSPR Chip with Microfluidics

**Figure 1:**
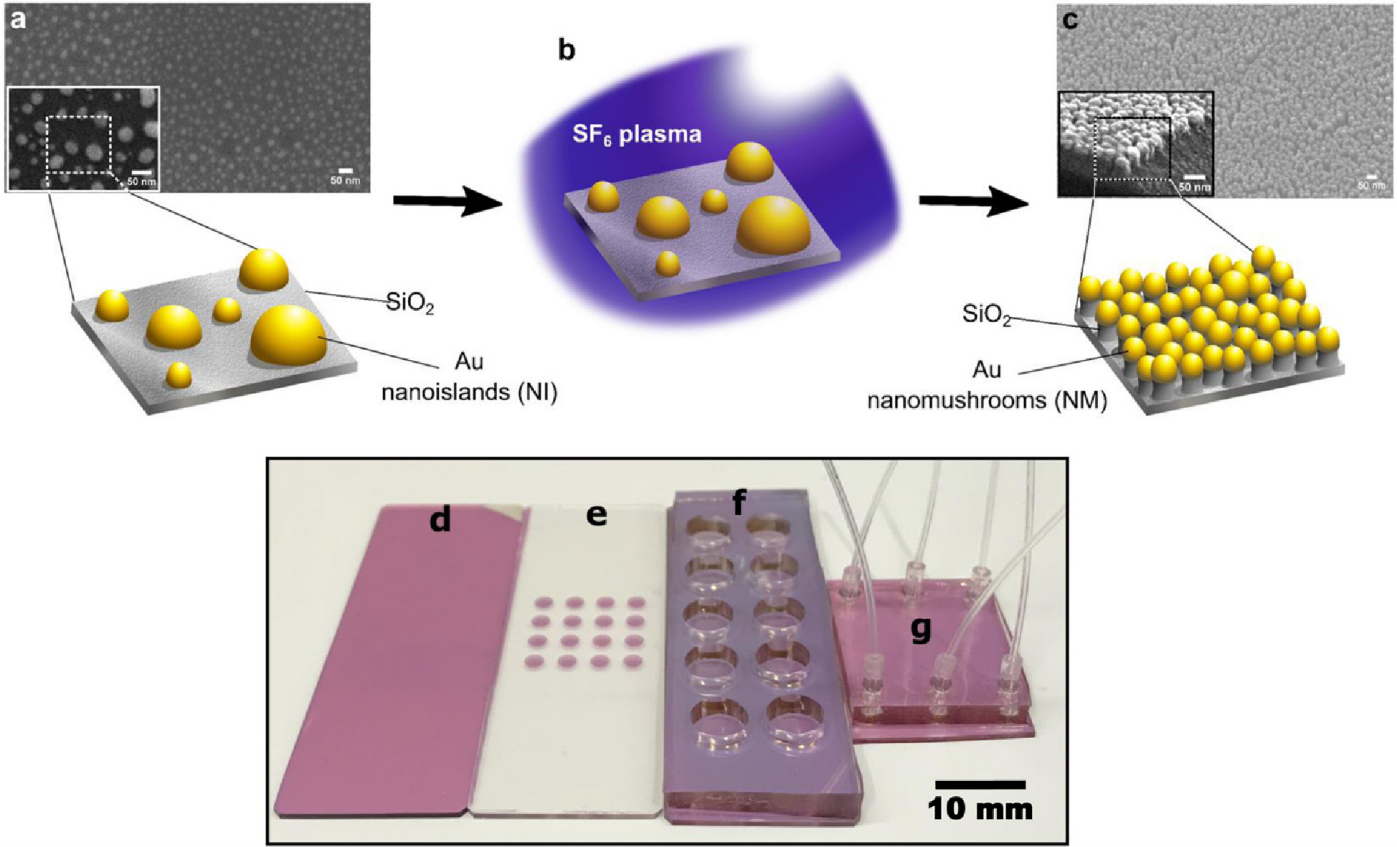
Fabrication of nanomushroom (NM) arrays in SF_6_ plasma. (a) Scanning electron microscopy (SEM) image and schematic representation of nanoislands (NI). (b) Schematic depicting an SiO_2_ substrate with NI inside an SF_6_ plasma chamber, where SF_*x*_ reactive ions etch out both SiO_2_ and Au. (c) SEM image and schematic of NM formed after the NI being exposed to reactive ionetching (RIE) inside SF_6_ plasma. The inset shows the cross section of the NM. The developed NM structures are 45–60 nm in total height with an average spacing of 7.96 ± 2.12 nm. (d–g) Examples of various configurations of NM substrates or integrated chips: (d) NM-coated glass slide of 2.5 cm × 7.5 cm (the pink color corresponds to the Au nanostructures); (e) spots of 3 mm circles for multiplex bioassay applications; (f) integrated polydimethylsiloxane (PDMS) wells on an NM substrate; (g) sealed device with PDMS microfluidic channels on an NM substrate connected with liquid delivery tubings. Reproduced with permission from ref (10).

In this section, we will delve into our past experiences in developing a simple LSPR chip and its integration with microfluidics.^35^ Additionally, we will explore potential avenues for refining the fabrication process. The following is a step-by-step protocol for creating an LSPR microfluidic chip:

1. *Selecting the ideal substrate for metal nanostructure fabrication:* Choose from standard options such as borosilicate glass slides, pure silicon dioxide (SiO_2_), silicon (Si), or silicon-based materials like silicon carbide or silicon nitride as dielectric substrates for crafting LSPR-active nanostructures. It is important to emphasize that the substrate choice significantly influences sensitivity, as demonstrated in our previous studies.^36^
2. *Substrate cleaning:* The selected substrate should be thoroughly cleaned. For instance, when working with pure silicon dioxide (SiO_2_) and silicon (Si), we employed the RCA (Radio Corporation of America) protocol, detailed as follows:^37,38^
  (a) *Removal of organic contaminants:* Prepare a solution with a 5:1:1 ratio of H_2_O:NH_4_OH: H_2_O_2_. To prepare this solution, start by adding NH_4_OH to deionized (DI) water. Heat the resulting mixture on a hot plate at 80 °C until noticeable bubbles form. At this point, introduce H_2_O_2_, which will significantly increase the bubble formation within the solution. While the solution continues to heat, immerse the substrate in it for 10 s to remove organic contaminants. After this step, thoroughly rinse the substrate with DI water and place it in a clean substrate holder.
  (b) *Removal of inorganic contaminants:* Prepare a solution with a 6:1:1 ratio of DI H_2_O:HCl:H_2_O_2_. First combine HCl with water (H_2_O) and heat the mixture on a hot plate at 80 °C until you observe bubbles forming in the solution. At this point, introduce H_2_O_2_, which will lead to a significant increase in bubble formation. While the solution continues to heat, immerse a precleaned substrate (already cleaned to remove organic contaminants) in the heated solution for 10 s to effectively eliminate inorganic contaminants. After this step, thoroughly rinse the substrate with DI water to complete the cleaning process, making it ready for its intended use. Note that many glass and silicon manufacturers now provide substrates that are already sufficiently clean, sometimes even polishing them before shipping. In such cases, the RCA cleaning protocol can be substituted with a milder cleaning method using acetone and isopropanol (IPA) as the cleaning agents.^39^ Initially, immerse the substrate in acetone and sonicate it for 5 min. Follow this with a thorough rinse using IPA to remove any residues or stains from the acetone cleaning process.^40^
3. *Thin film deposition:* After completing the cleaning process, apply a gold film with a thickness of less than 5 nm to the meticulously cleaned substrate. It is important to note that the thickness of this gold film directly impacts the size and spacing of the resulting nanostructures. While thicknesses up to 10 nm can yield nanoparticles,^41^ for optimal sensitivity when creating NM structures, we recommend a film thickness of up to 5 nm.
4. *High-temperature annealing:* The selected substrate, featuring a deposited thin film of gold, undergoes annealing at a temperature of 560 °C for a duration of 3.5 h within a hot furnace. Note that the temperature of annealing is different for different substrates/LSPR active material as it mainly depends on the critical temperature of the material with which the chip is developed. Following this annealing process, plasmonic gold nanoislands form on the substrate, displaying a distinctive pinkish coloration. At this stage, we have successfully developed a nanoisland (NI) LSPR chip.
5. *Etching:* The NI LSPR chip is now exposed to inductively coupled plasma reactive ion etching (ICP-RIE) technique with SF_6_ gas to convert the nanoisland (NI) into nanomushroom (NM) configuration. In our specific study, the ICP-RIE instrument’s internal chamber pressure was set to 10 mTorr, with a flow rate of 45 sccm (standard cubic centimeters per minute) for SF_6_. The radio frequency (RF) power coil and RF bias coils were set to 150 and 10 W, respectively, while maintaining a plasma chamber temperature at 5 °C. The entire reactive ion etching process lasted for 5 min. At this stage the NM-based LSPR chip is successfully developed on the selected substrate. For a comprehensive understanding of the fabrication process and various configurations of the NM-based LSPR chip, please refer to Figure 1.

The subsequent step in establishing the LSPR microfluidic chip involves the creation of microfluidic structures and their integration with the NM-based LSPR chip.

1. *Master mold fabrication:* Generate a master mold with the desired microchannel pattern by utilizing a photoresist such as diazonaphthoquinone (DNQ) or SU-8 on a silicon substrate. Following this, the master mold is treated with trichloro(1*H*,1*H*,2*H*,2*H*-perfluorooctyl)silane, a critical surface chemistry treatment aimed at reducing adhesion between polydimethylsiloxane (PDMS) and the master mold.
2. *Development of PDMS structures:* Pour a well-mixed blend of PDMS and its curing agent, typically in a 10:1 ratio, onto the master mold. After degassing, bake the mixture at 60 °C for 24 h to ensure complete polymerization of PDMS. Then, delicately peel the solidified PDMS away from the master mold.
3. *Fluidic connections:* Create openings for fluid inlet and outlet tubing; a biopsy punch is employed.
4. *Bonding:* The PDMS channels, with their specific patterns, are prepared for attachment to a glass or silicon substrate containing gold nanostructures (i.e., NM LSPR chip). Both the PDMS and LSPR chip undergo O_2_ plasma exposure in a plasma chamber. This O_2_ plasma treatment generates transient −OH groups on the PDMS surface, facilitating a robust bond with OH groups on the glass or silicon. The bonding effectiveness can be strengthened by heating the bonded assembly at 60 °C for 1 h. After cooling the bonded PDMS device to room temperature, it is ready for utilization in fluidic experiments.

**Figure 2:**
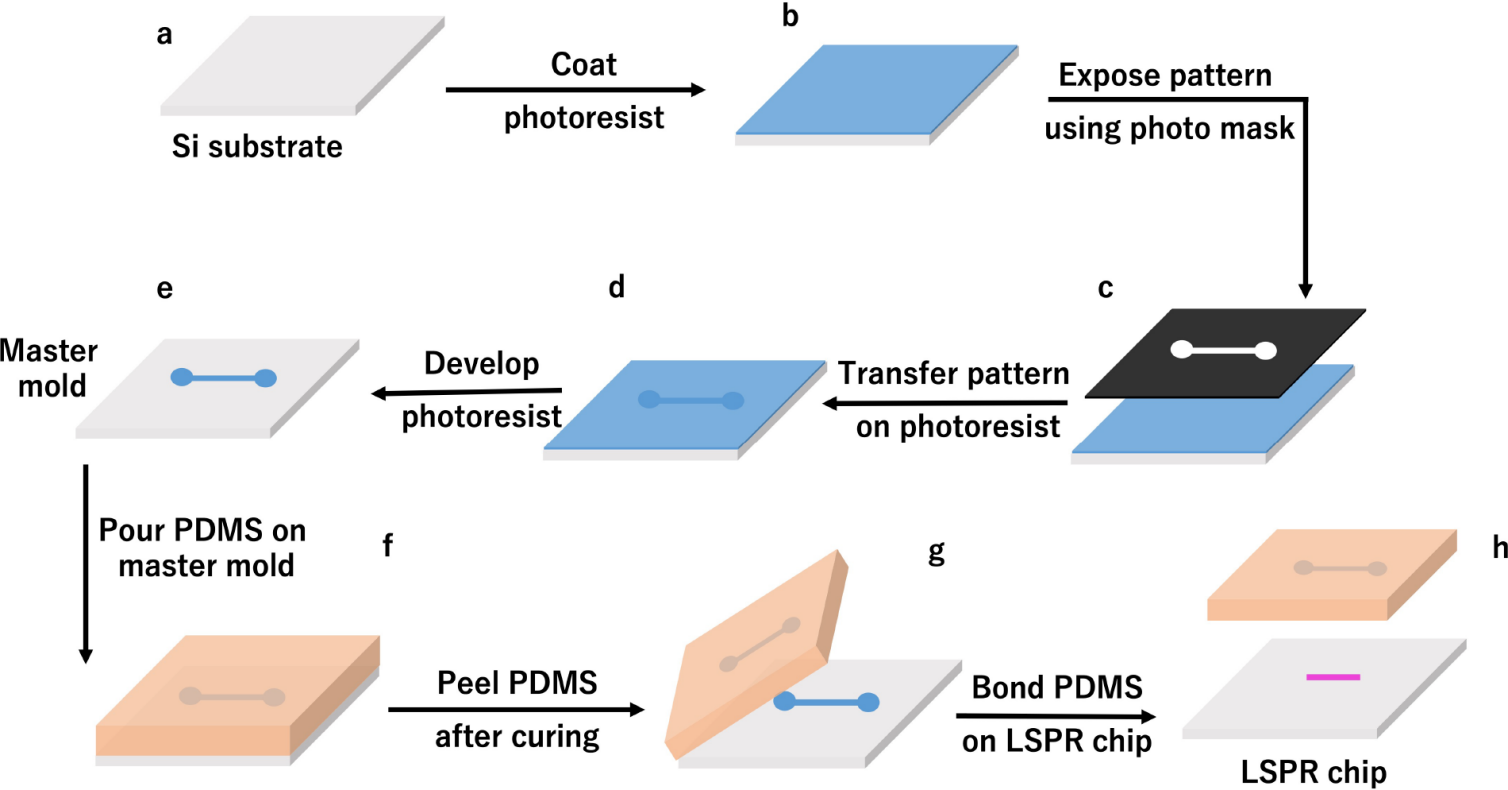
Soft lithography steps: (a) Si substrate, (b) coating of photoresist on Si substrate, (c) exposure of photoresist to micropatterns, and (d) transfer of micropatterns onto the photoresist. (e) Development of the master mold. (f) and (g) represent the transfer of micropatterns from the mold to PDMS, and (h) LSPR and PDMS substrates ready for bonding. Reproduced with permission from ref (35). Copyright 2018 Elsevier.

Finally, assembly of the LSPR microfluidic chip is completed. The detailed fabrication steps for creating the PDMS template with microfluidic channels and its subsequent bonding to the LSPR chip are visually explained in Figure 2.

It should be noted that PDMS can also be replaced with PMMA (poly(methyl methacrylate)) to fabricate microchannels. Fabriating microfluidic–nanoplasmonic devices with PMMA entails the design of micropatterns using AutoCAD, followed by the printing of layers onto a PMMA substrate using a laser cutter or micromilling machine and subsequently assembling them to form a fully functional microfluidic device.

## Biosensing Using Microfluidic LSPR Chips

**Figure 3:**
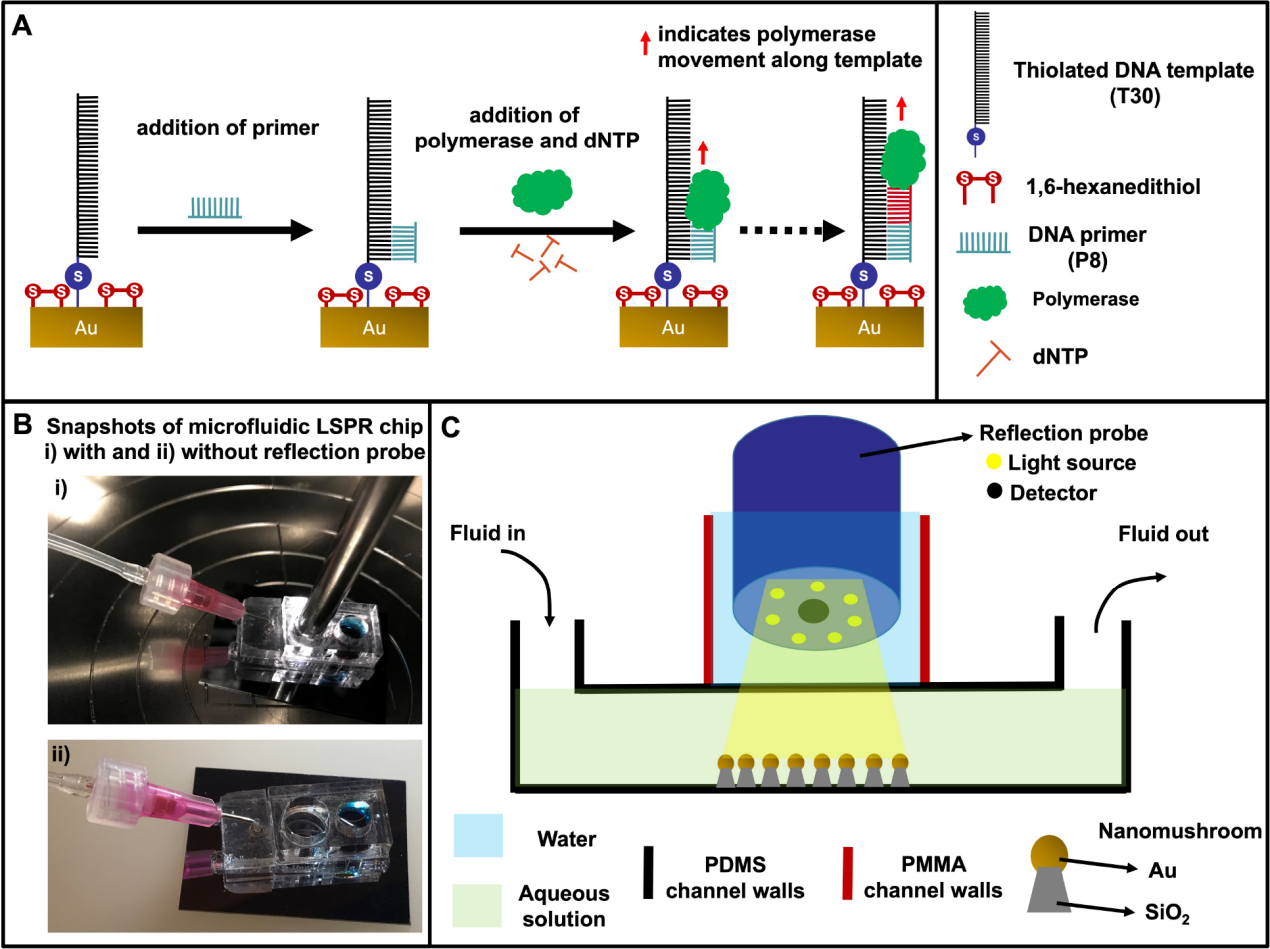
LSPR DNA sensing microfluidic setup (A) Reaction scheme on a gold (Au) LSPR substrate, involving an immobilized ssDNA template (T30) with HDT, addition of primer sequence P8, and Klenow fragment of DNA polymerase along with dNTPs. Polymerase catalyzes the formation of the complementary DNA strand by assembling dNTPs from the surrounding media. (B) Snapshots of a LSPR microfluidic chip, in operation with indented reflection probe (i) and without (ii). In both cases the fluid inlet reservoir and the outlet tubing are shown, and (C) shows a schematic of the microfluidic nanoplasmonic chip consisting of the bottom nanoplasmonic substrate, a PDMS, and a poly(methyl methacrylate) (PMMA) substrate. Reproduced with permission from ref (11). Copyright 2019 Elsevier.

**Figure 4:**
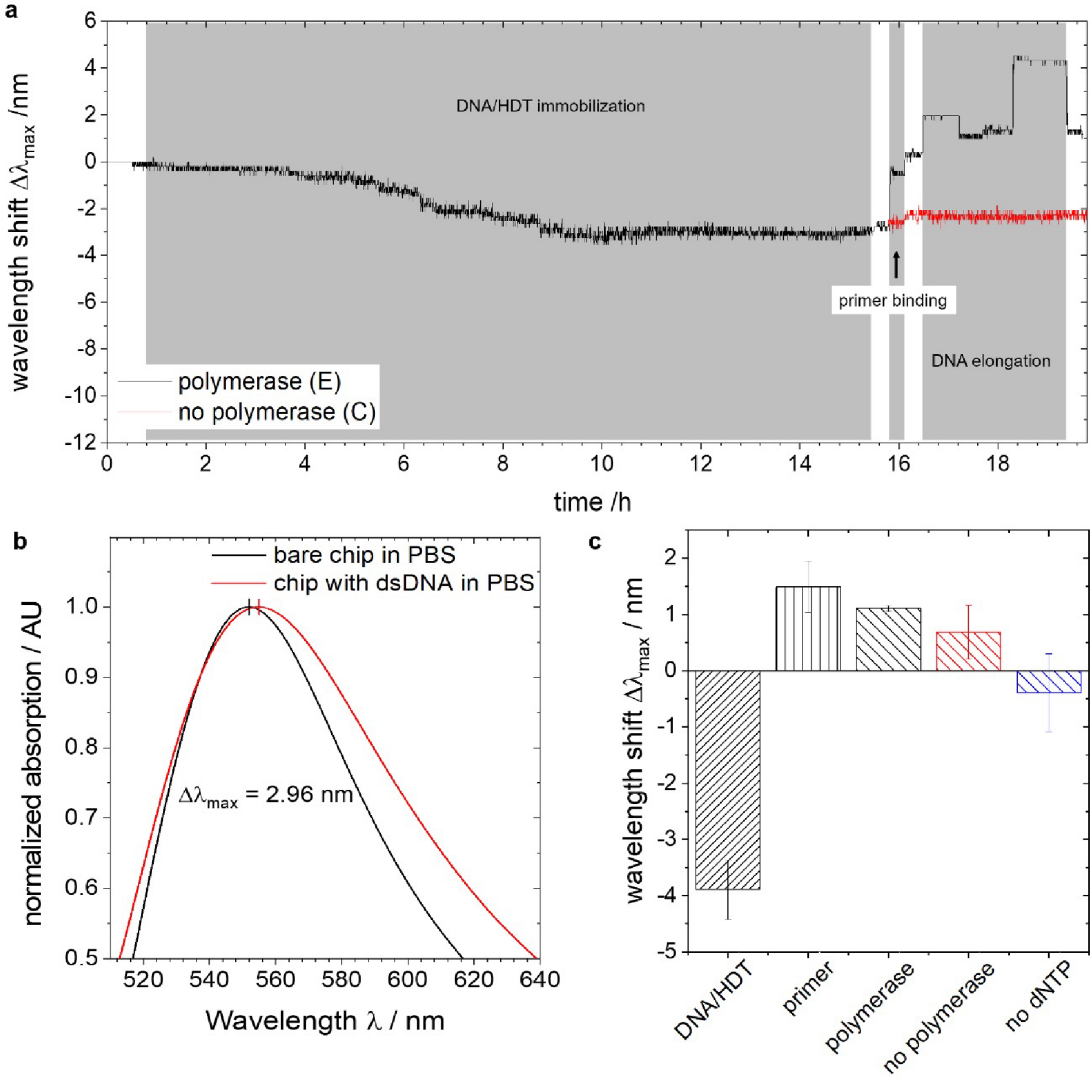
Label-free real-time DNA/HDT immobilization and polymerase activity monitoring using LSPR measurements. (a) Real-time sensogram showing the shift in the maximum wavelength of the reflected light during immobilization of DNA and HDT, primer binding, DNA elongation, and intermediate washing steps. (b) Sample reflection spectra of bare microfluidic chip and the chip with ds30-mer showing a total wavelength shift of 2.7 nm. (c) Mean wavelength shifts from each step, calculated from 6 polymerase reactions and 3 controls (no polymerase and no dNTPs) experiments. Error bars represent the standard error of the mean. The polymerase versus “no dNTP” is significant with *p* < 0.05. Reproduced with permission from ref (11). Copyright 2019 Elsevier.

Here, we present a microfluidic LSPR chip designed for real-time, label-free DNA molecule detection and analysis.^11^ The microfluidic chip is initially constructed with a PDMS microfluidic channel, which is subsequently bonded to the LSPR chip. This LSPR chip is characterized by NM nanostructures, comprising silicon dioxide stems (40 nm) and gold caps (22 nm), with an average spacing of 19 nm. To enable effective spacing and surface distribution of specific single-stranded DNA (ssDNA), denoted as T30, on the LSPR substrate, hexanedithiol (HDT) molecules are introduced in a 1:1 molar ratio with the DNA. This prepares the substrate for subsequent molecular interactions. A DNA primer, identified as P8, is then attached to the existing DNA strands on the LSPR chip. The crucial step involves the action of DNA polymerase, an enzyme catalyzing the elongation of DNA strands. This process involves the sequential assembly of nucleotides from the surrounding fluid, effectively creating a complementary DNA strand. Further details regarding the sensing mechanism and sensor response are elucidated in Figure 3 and Figure 4.

Importantly, all of these molecular interactions and reactions occur within the confines of the microfluidic LSPR chip. The real-time nature of this detection method enables researchers to observe and monitor each step of the intricate reaction as it unfolds, all at room temperature. For instances, from results shared in Figure 4, we can observe a shift of 4.19 ± 0.48 nm upon polymerization of double-stranded DNA. These wavelength shifts are in close agreement with the theoretical expected wavelength shifts of 3.24 nm for a LSPR sensor with a sensitively of 54 ± 6 nm, when used for interrogation of 0.06 units change in refractive index. Insights into dynamics of individual steps of primer attachment, elongation by polymerase, and its release can also be observed in the real time (Figure 4). However, without additional extensive validation the reaction steps using other complementary techniques (such as quartz crystal microbalance) or testing other DNA systems, only qualitative conclusions related to the completion of the reaction can be drawn from the results. It should also be noted that the wavelength shifts are computed for each reaction step relative to the signal change observed in the preceding step. The overall wavelength shifts throughout the reaction are determined relative to the beginning of the reaction.^11^ This detection process is entirely label-free, eliminating the need for external markers or labels. Ultimately, we establish a correlation between the sensor response and the quantities of DNA and HDT molecules immobilized on the surface. This correlation allows for a comprehensive and detailed analysis of DNA-related reactions, providing valuable insights into molecular-level processes through the microfluidic LSPR chip. A general qualitative comparison of LSPR-based microfludic chip versus the other microfluidic chips in the literature is also shared in Table 2.

**Table 2:** Qualitative Comparison of LSPR-Based Microfluidic Chips versus Other Microfluidic Chips

feature	LSPR-based microfluidic chips	other microfluidic chips
sensitivity	high	dependent on specific design/approach
label-free detection	yes	dependent on specific methodology
multiplexing capabilities	yes	present in various designs
integration with nanoparticles	yes	possible with certain designs/approaches
signal-to-noise ratio	high	dependent on design and implementation
fabrication complexity	moderate	varies (e.g., PDMS, glass)
cost	moderate to high	dependent on materials and fabrication
transduction mechanisms	optical (LSPR)	various (e.g., electrochemical, fluorescence)
real-time analysis	yes	feasible with suitable designs
sample volume	low	dependent on chip design and application
resolution	high	dependent on design and detection method
compatibility	biocompatible	dependent on materials and applications
sensing range	wide	dependent on detection mechanism
on-chip automation	possible	feasible with appropriate designs
portability	yes	dependent on size and components
reusability	limited	feasible with specific designs/materials
power consumption	low	dependent on design and operation
environmental sensitivity	moderate	dependent on materials and conditions
stability	high	dependent on materials and fabrication
scalability	moderate	feasible with suitable designs
compatibility with lab-on-a-chip systems	yes	feasible with appropriate designs
detection limit	low	dependent on detection method and design
ease of integration with external devices	moderate	feasible with suitable interfaces
complexity of data analysis	moderate	dependent on detection method and design
compatibility with microfluidic pumps	yes	feasible with compatible designs
ability to handle multiple sample types	yes	dependent on chip design and application
compatibility with microscopy	yes	feasible with suitable designs
long-term stability	high	dependent on materials and conditions

## Specific Gravity Sensing of Biofluids

The measurement of biofluid specific gravity is a critical parameter that can be directly addressed by using LSPR chips. Assessing the specific gravity of biological fluids such as urine, sputum, blood plasma, and serum plays a pivotal role in clinical diagnostics.^42−44^ Specific gravity assessment aids in evaluating the concentration of solutes in these fluids, offering insights into hydration levels, kidney function, and potential disorders. In the case of sputum, specific gravity analysis aids in the evaluation of respiratory conditions.^45^ Meanwhile, deviations observed in blood plasma and serum specific gravity may signal dehydration or abnormal solute concentrations, assisting in the diagnosis of metabolic disorders or renal issues.^46^

**Figure 5:**
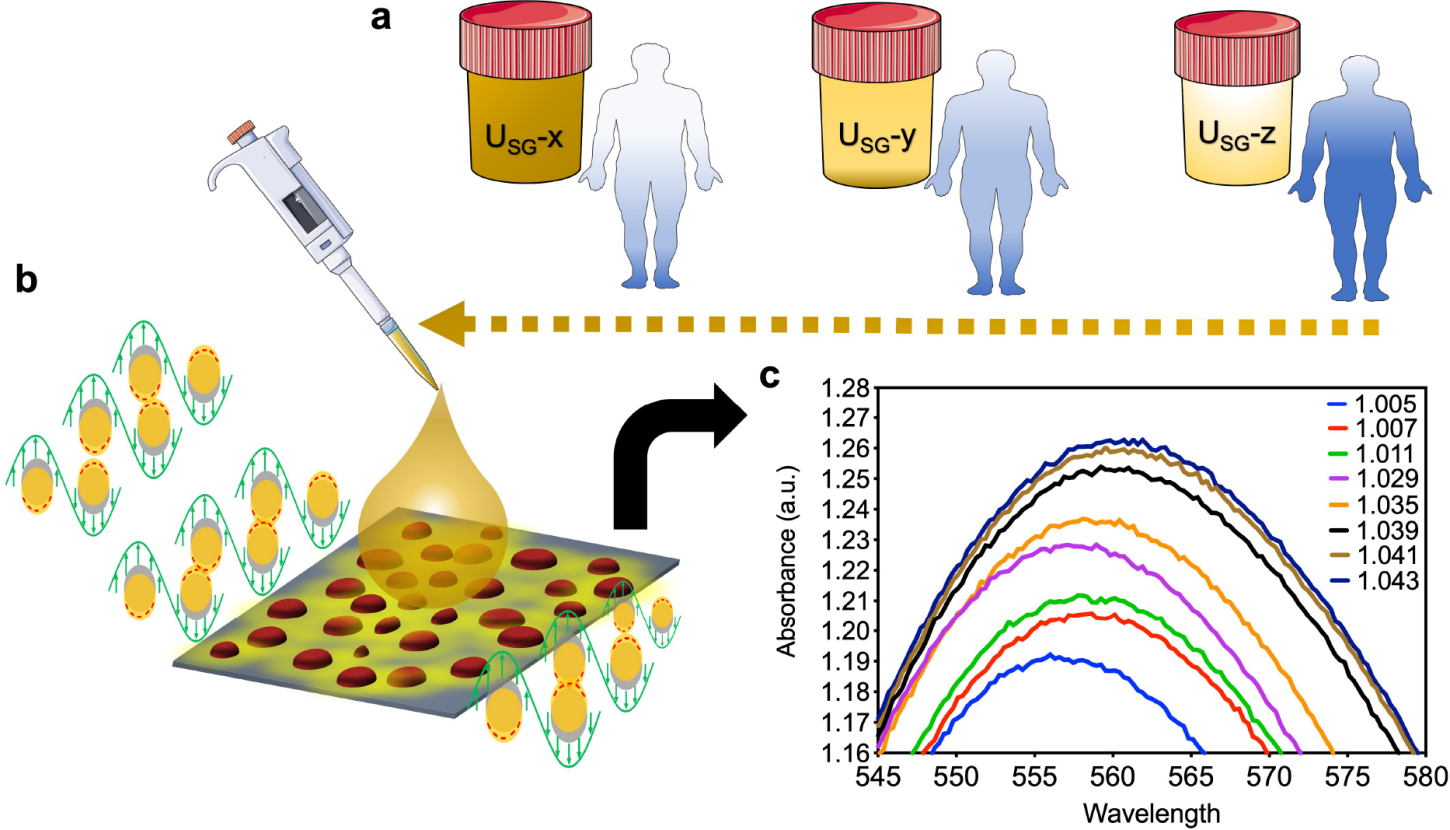
Urine specific gravity measurement. (a) Three individuals exhibiting distinct hydration levels, each reflected in varying urine specific gravity. (b) Schematic representation of the LSPR measurement process. (c) LSPR spectra, showcasing absorbance and wavelength shifts corresponding to changes in urine specific gravity. Adapted with permission under a Creative Commons CC-BY 4.0 license from ref (47). Copyright 2024 Wiley.

In our research, we have conducted specific gravity measurements of urine, covering a range from 1.005 to 1.043.^47^ These measurements were made using real human urine samples in an experiment where urine was collected from the donor before and after running a 10 km distance (to assess the hydration conditions). The collected urine was then subjected to the LSPR chip made of gold nanoislands, and we observed a red-shift when the darker color urine (higher specific gravity) was tested using the LSPR chip. The sensitivities of the chip were found to be 79.21 nm per urine specific gravity unit. Further details are provided in Figure 5. This precise measurement of specific gravity serves as a reliable indicator for assessing hydration levels, with variations indicating potential dehydration or overhydration. Furthermore, it plays a crucial role in evaluating kidney function, as deviations in specific gravity may reveal renal insufficiency. Beyond that, this diagnostic parameter proves invaluable in the monitoring and early detection of urinary tract infections, kidney stones, and fluid retention, facilitating prompt intervention and tailored treatment plans.^48^

**Figure 6:**
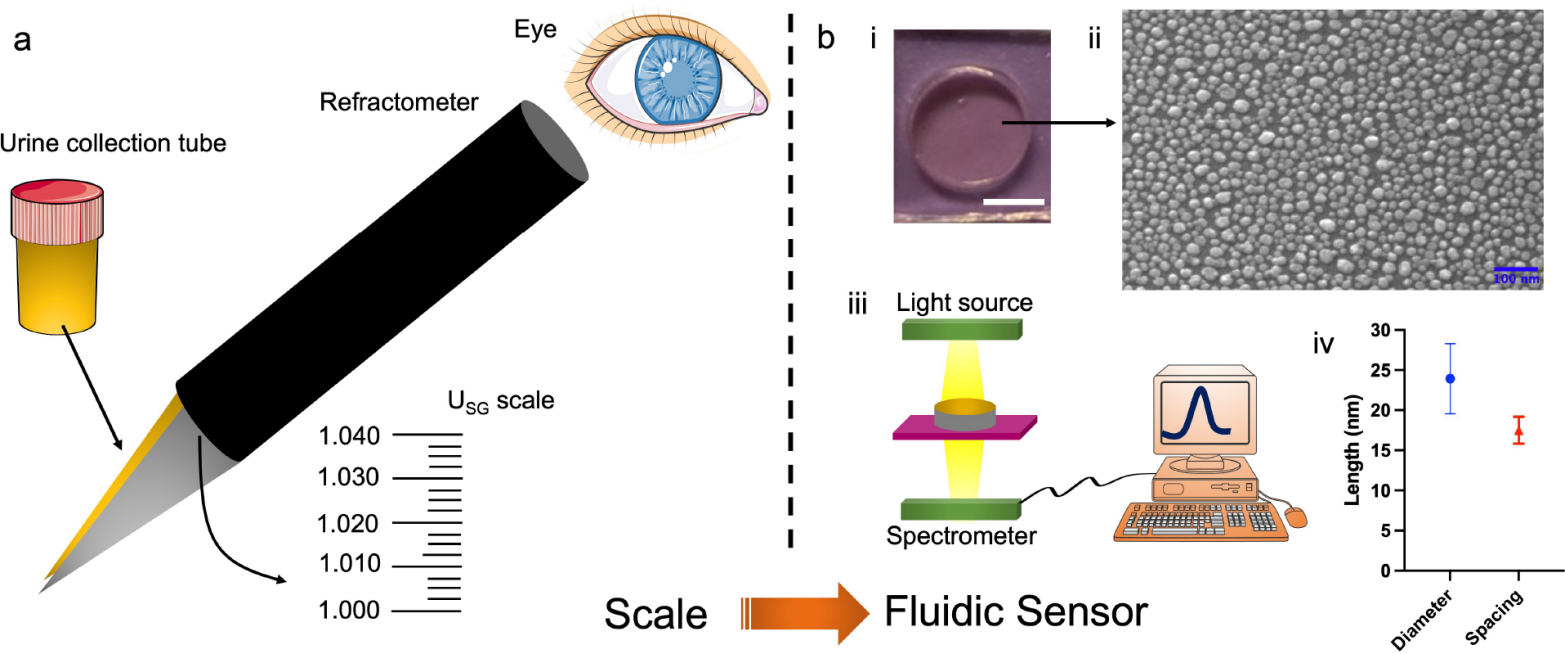
Scale to fluidic sensor: (a) Schematic of a refractive index scale for the measurement of urine parameters which involves observation using the human eye. The schematic also shows a urine collection tube. The arrows indicate the location of the scale (drawn not to the scale) and the location where urine is added on the refractometer. (b) Part i shows a picture of the LSPR sensors, where the length of the scale is 1 cm. Part ii shows a scanning electron microscopy image of the LSPR sensor surface. Part iii shows measurement of the schematic. Part iv reveals the mean diameter and spacing of the nanoislands on the LSPR sensor surface. Reprinted with permission under a Creative Commons CC-BY 4.0 license from ref (47). Copyright 2024 Wiley.

From the sensor’s perspective, the LSPR sensor offers a distinct advantage over traditional specific gravity measurement tools like refractometers, which most rely on subjective observations susceptible to variation among individuals (a scale is read by the human eye; see Figure 6, showing the evolution of a traditional specific gravity measurement scale to a fluidic sensor). While there are refractometers described in the literature^49^ that do not rely on direct visual reading of measurements, such sensor types (compared to traditional refractometers) have typically not exclusively found clinical applications such as for urine specific gravity measurement. LSPR mitigates such potential errors or discrepancies by directly displaying sensor responses, eliminating the reliance on human interpretation. Beyond its sensitivity and selectivity, LSPR sensors provide the added benefit of easy storage or transmission of data to a database. This facilitates advanced functionalities, including its integration with artificial intelligence, enhancing the overall efficiency and capabilities of the measurement system.

## Deterministic Particle Assembly on LSPR Chips: Toward Pumpless Flow Control

Controlled particle assembly plays a pivotal role in both materials science and analytical biology.^50,51^ Accurate particle arrangements result in advanced materials that exhibit customized optical and electronic properties. In the realm of analytical biology, this capability enables the assembly of bioentities, serving a multitude of scientific purposes, including biosensing and the development of organ-on-chip systems.^52,53^ In our research, we have successfully demonstrated particle assembly using LSPR chips for micrometer-sized silica particles.^32^ This process is initiated by the formation of laser-induced hot spots on LSPR chips, resulting in the generation of microbubbles that guide the assembly of the particles. While thermoplasmonic flow and microbubble formation are observed with SPR substrates as well, it is important to note that the exclusive capability of particle assembly is unique to LSPR chips. This distinction is attributed to the presence of nanoscale air gaps and the dissipation of electron energy within LSPR nanostructures.

**Figure 7:**
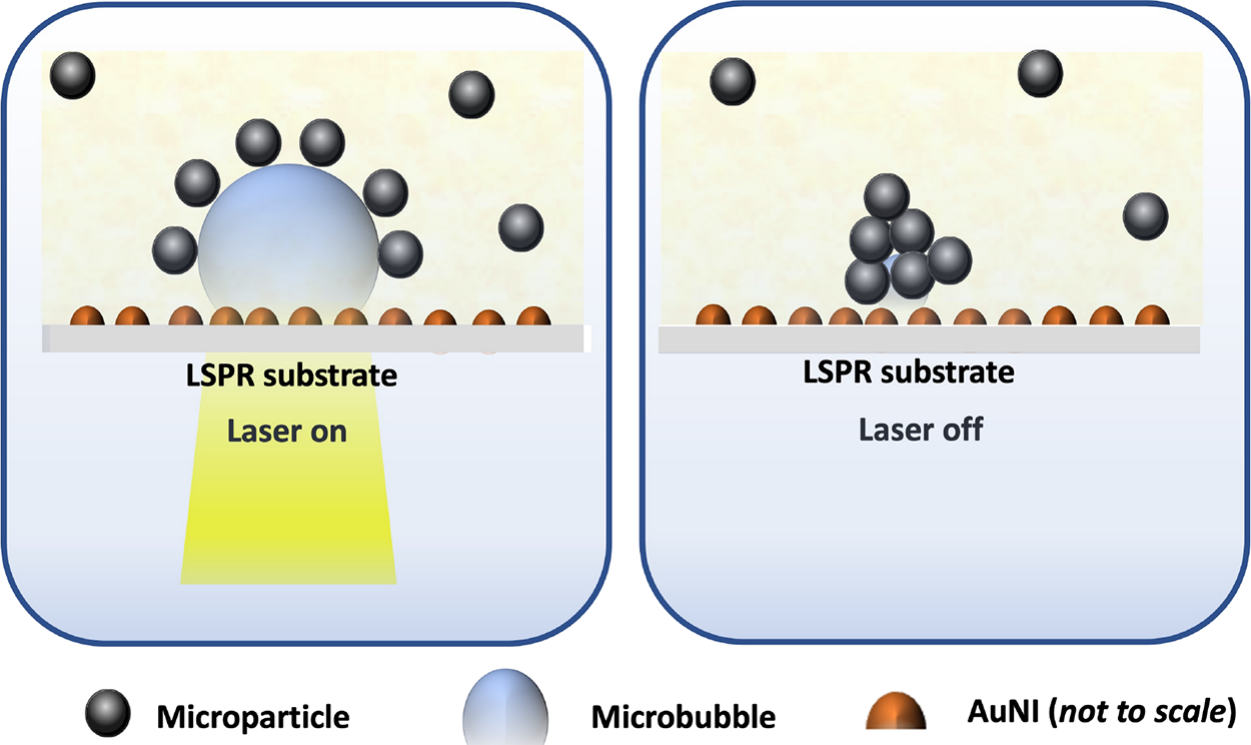
Self-assembly of microparticles on LSPR chips.

By carefully adjusting parameters, such as laser power, circular laser sequence radius, and particle concentration, we gain precise control over the particle count and the assembled structure. This framework firmly establishes LSPR chips as a versatile platform for light-driven particle assembly, with further insights provided in Figure 7.

The formation of clusters can be elucidated by the solvent evaporation process induced by heating the nanostructures with the laser. This heating generates a temperature gradient within the liquid as the bubble expands. Consequently, the growing bubble induces a convective flow of fluid toward itself, effectively bringing the particles closer to the surface of the microbubble. When we disregard inertia and gravitational effects, the motion of these particles can be described by eq 3:
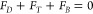
3where *F*_*D*_ is the drag force, *F*_*T*_ is the thermophoretic force, and *F*_*B*_ is the Brownian force. In our system, *F*_*D*_ dominates the other two forces. The Peclet number, the ratio between flow and Brownian time scales, is on the order of 1 × 10^2^ for our experiments. Additionally, the thermophoretic velocity is 10^–6^ m/s, which is 1 order of magnitude lower than the translational velocity (2 × 10^–5^ m/s) of the particles in our experiments.

**Figure 8:**
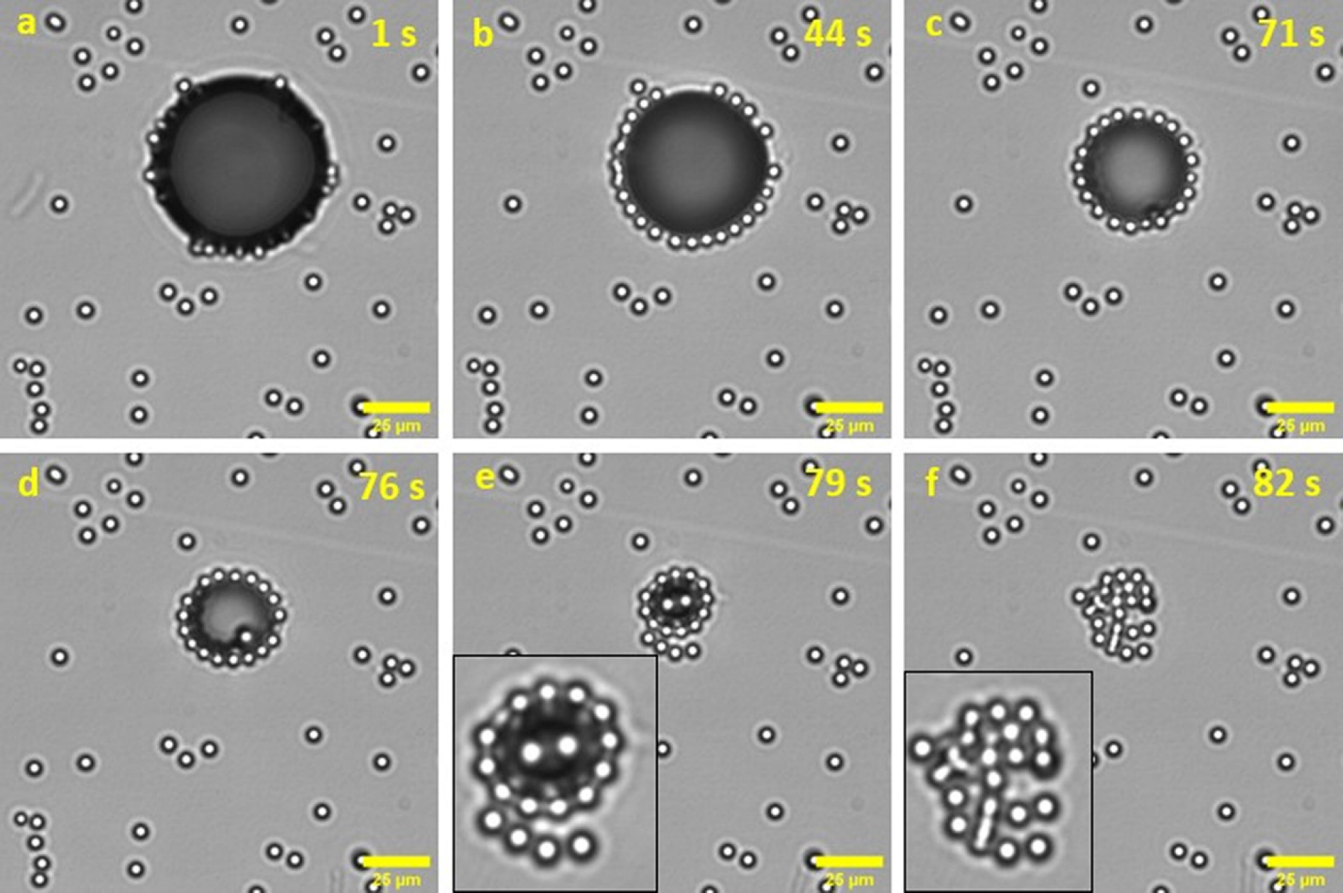
Decay of the bubble radius upon the laser removal. The pictures are captured at (a) 1, (b) 44, (c) 71, (d) 76, (e) 79, and (f) 82 s after removing the laser beam. The scale bar is 20 μm. The concentration of the dispersion and the size of the laser sequence used in these experiments were 0.04 g/L and 10 μm, respectively. Reproduced with permission from ref (32). Copyright 2021 Elsevier.

When particles reach the vicinity of the bubble, they are primarily influenced by the dominant drag force, which notably does not cause the bubble to collapse. This intriguing phenomenon can be attributed to the favorable ratio between the drag force generated by particle velocity and the gravitational force acting on the particles, roughly on the order of 1 × 10^–1^. Consequently, the particles tend to accumulate at the contact line between the substrate and the bubble interface. Once pinned at this interface, these particles accompany the bubble during the condensation process and ultimately coalesce into a cluster when the bubble eventually collapses. The self-assembly process is visually depicted in Figure 8, illustrating the gradual decay of the bubble radius upon laser removal.

One intriguing application of this concept involves the exploration of cell–cell interactions within a microfluidic device, obviating the need for an external pump.^54,55^ In this scenario, cells suspended in a solution could be strategically assembled and transported from one location on a chip to another by using a microbubble. This process facilitates the mixing of cells with other biomolecules present on the chip before the bubble ultimately collapses. The microbubble’s specific density could also enable it to selectively lift particular cells and transport them to different locations on the chip. Notably, since the substrate is plasmonic, the same laser used for bubble generation and transport can also serve as a tool for detecting LSPR signatures or changes in refractive index resulting from the assembled species. This dual-purpose utilization of the laser enables both sensing and pump-free fluid manipulation, encompassing activities such as fluid mixing and transport.

With regard to application, the integration of particle assembly techniques with LSPR chips presents a new opportunity in bio/chemical sensing capabilities. For instance, by leveraging precise control over the particle arrangement, researchers can tailor sensing platforms with superior sensitivity, selectivity, and dynamic range. This approach enables the development of multiplexed sensing systems capable of the simultaneous detection of multiple analytes within a single assay, expanding the scope of applications in fields such as medical diagnostics, environmental monitoring, and food safety. Moreover, the dynamic nature of particle assembly on LSPR chips facilitates real-time modulation of sensor performance in response to environmental cues or analyte interactions, enabling on-demand tuning of the sensitivity and specificity. Integration with microfluidic systems further enhances sensor functionality by enabling pumpless flow control and precise sample manipulation, leading to improved mass transport kinetics and reduced sample volumes. Together, these advancements herald a new era of biosensing technology, where the synergy between particle assembly and LSPR chips unlocks novel possibilities for the development of versatile, high-performance sensors with broad-ranging applications in biomedical, environmental, and industrial settings.

## Exploring the Potential of LSPR Chips in Interfacial Science

LSPR sensors can be employed in examining the interfacial phenomena at both solid–liquid and liquid–liquid interfaces.^56−58^ This sensitivity stems from LSPR’s ability to detect changes in the refractive index of nanostructures situated within distances of 5–10 nm from the surface. This specific range aligns with the realm where interfacial effects hold significant sway, rendering the LSPR highly relevant for exploring a wide array of applications. These applications span from gaining insights into fluid rheology to detecting complex phenomena like liquid slips.^59,60^

LSPR sensors offer a valuable means to explore various liquid–liquid interactions, with emulsification serving as a prominent example.^61,62^ Emulsification is the process of dispersing one liquid into another in the form of small droplets and is encountered in everyday products such as salad dressing and mayonnaise, which are typical oil-in-water type emulsions. It is worth noting that certain surfactants, such as egg in mayonnaise, are used as stabilizers in such mixtures to reduce interfacial tension between water and oil components.

Leveraging LSPR sensor sensitivity to changes in the refractive index at the oil–water interface can be invaluable for scrutinizing the dynamic interplay between these substances, offering potential insights into optimizing interfacial tension and, consequently, refining taste. Additionally, LSPR can facilitate the study of fluid parameters crucial for interfacial rheology, including the density and viscosity of the liquid–liquid interface.^63,64^ This is due to the refractive index’s dependence on factors like density and viscosity in the context of the liquid–liquid interface.

The versatility of LSPR can extend to examining flow effects, such as Marangoni effects, where variations in surface tension induce fluid flow, resulting in the movement of one liquid over the other.^65^ This offers an opportunity to investigate two vital parameters: the refractive index of moving fluids compared with stationary liquids and the quantification of surface tension using optical signatures of plasmons at the liquid–liquid interface. LSPR methods can also provide a useful platform for exploring the complexities of viscoelastic flow instabilities and elastic turbulence.^66,67^

**Figure 9:**
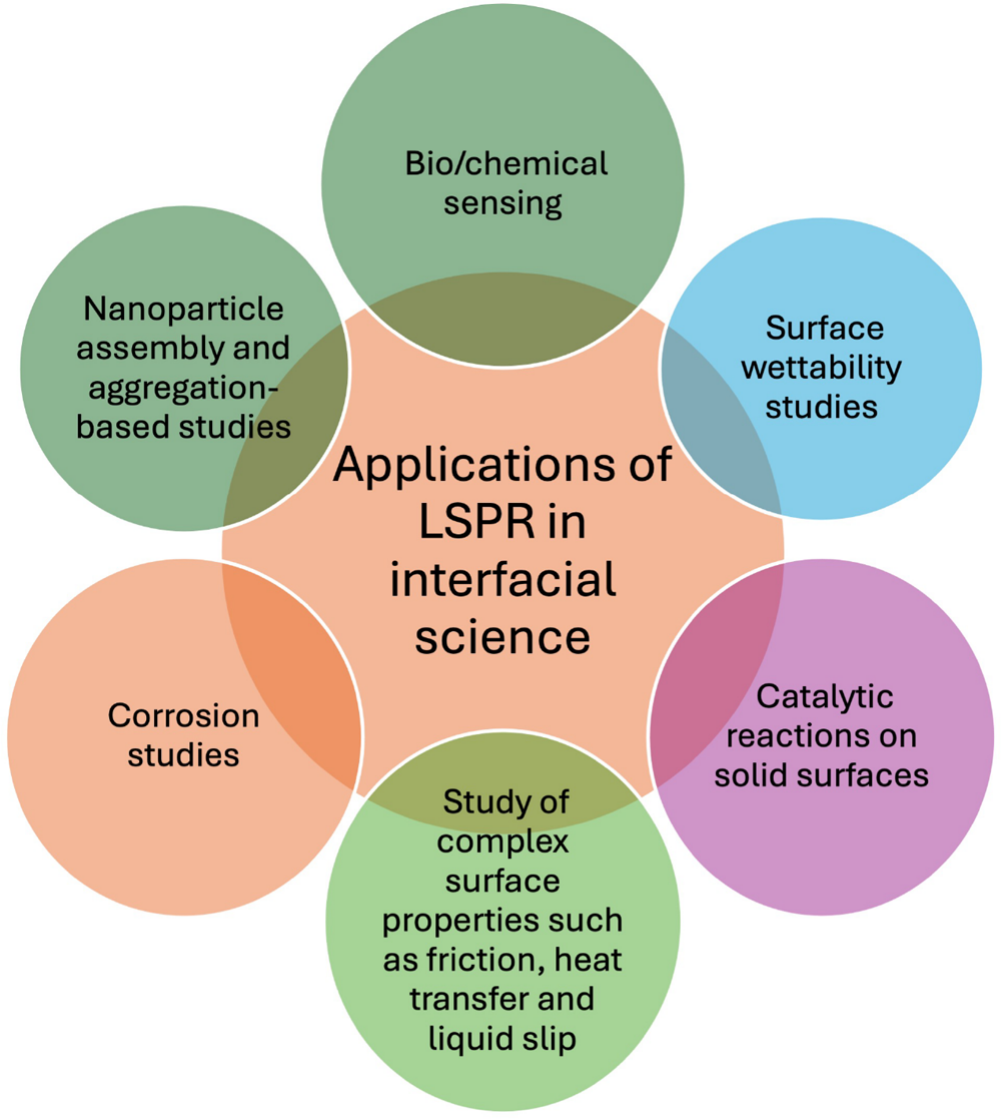
Applications of LSPR interfacial science.

Exploring solid–liquid interfaces is crucial for a comprehensive understanding of fluid flows, demanding surface-sensitive experimental techniques such as atomic force microscopy and nonlinear optical methods. In this context, LSPR provides a unique opportunity to investigate solid–liquid interfaces through high-frequency resonating nanostructures. The configuration of LSPR nanostructures can be adjusted to create surfaces with different wettabilities, ranging from hydrophobic to hydrophilic features. These varied wetting conditions on solid surfaces offer a distinctive platform for fluid interactions, leading to the generation of friction forces, shear stress, and liquid slips at the solid–liquid interface.^68,69^ These forces directly influence the properties of the nanostructures, which can be elucidated through absorption spectroscopy to identify the associated LSPR resonances. Another area of investigation is the bacteria biofilms.^9,69^ LSPR can be useful to investigate dynamics of how the biofilm interacts with the solid surfaces, revealing properties such as adhesive forces and viscoelastic properties of the film. Some other applications where LSPR can be extended in the near future are also shared in Figure 9.

## Conclusions and Future Prospects

The future of LSPR sensors promises significant advancements across diverse fluidic domains, particularly in the interplay between electrical plasmons and fluidic interfaces. This emerging area holds great potential for developing novel measurement techniques at the solid/liquid interface and uncovering new scientific insights. To fully realize this potential, further research is needed to establish a synergy between electromagnetic fields and fluidic environments.

One of the central challenges for future LSPR sensing lies in designing sensor chips with enhanced sensitivity and stability. Advancements in chip technology are essential to ensure precise and reliable measurements in dynamic and complex fluidic environments. This necessitates the refining of the structural and material composition of LSPR chips, potentially leading to more selective sensors. Additionally, the discovery and integration of new materials will expand the capabilities of LSPR sensors, allowing for tailored designs to optimize the performance in various fluid dynamics experiments.

In the realm of bio/chemical sensing, a deeper understanding of how LSPR sensors interact with biological entities is imperative. Isolating the effects of fluid flow is crucial for accurate interpretation in sensing applications, leading to more precise and reliable biosensor responses, with applications spanning medical diagnostics to environmental monitoring.

The future of LSPR sensing will also witness a surge in sophisticated simulation techniques, enabling researchers to model and predict LSPR behaviors under diverse fluid flow conditions. This predictive capability will provide valuable insights into sensor responses before experimental implementation and results interpretation.

Efficiently transferring LSPR sensor technology from research settings to practical applications is paramount. Bridging the gap between academia and industry will facilitate the integration of LSPR sensors into real-world scenarios ranging from healthcare to industrial processes. While miniaturization has enabled portable LSPR sensors, striking the right balance between sensitivity and portability remains crucial. Future developments should aim to optimize sensor size without compromising performance, particularly for on-site and field applications, where portability is a key consideration.

In summary, the future of LSPR sensors will be characterized by interdisciplinary collaborations, technological breakthroughs, and a deeper understanding of the interplay among fluid physics, interfacial science, materials, and biological entities. As researchers continue to push the boundaries of LSPR technology, its transformative impact on diverse fields will become increasingly evident, opening new avenues for exploration and innovation.

## References

[ref1] Willets K. A.; Van Duyne R. P. Localized surface plasmon resonance spectroscopy and sensing. Annual Reviews of Physical Chemistry 2007, 58, 267–297. 10.1146/annurev.physchem.58.032806.104607. 17067281

[ref2] Mayer K. M.; Hafner J. H. Localized surface plasmon resonance sensors. Chem. Rev. 2011, 111, 3828–3857. 10.1021/cr100313v. 21648956

[ref3] Li J.; Cushing S. K.; Meng F.; Senty T. R.; Bristow A. D.; Wu N. Plasmon-induced resonance energy transfer for solar energy conversion. Nat. Photonics 2015, 9, 601–607. 10.1038/nphoton.2015.142.

[ref4] Doiron B.; Mota M.; Wells M. P.; Bower R.; Mihai A.; Li Y.; Cohen L. F.; Alford N. M.; Petrov P. K.; Oulton R. F.; Maier S. A. Quantifying figures of merit for localized surface plasmon resonance applications: a materials survey. ACS Photonics 2019, 6, 240–259. 10.1021/acsphotonics.8b01369.

[ref5] Hammond J. L.; Bhalla N.; Rafiee S. D.; Estrela P. Localized surface plasmon resonance as a biosensing platform for developing countries. Biosensors 2014, 4, 172–188. 10.3390/bios4020172. 25587417 PMC4264378

[ref6] Funari R.; Chu K.-Y.; Shen A. Q. Detection of antibodies against SARS-CoV-2 spike protein by gold nanospikes in an opto-microfluidic chip. Biosens. Bioelectron. 2020, 169, 112578 10.1016/j.bios.2020.112578. 32911317 PMC7467868

[ref7] Qiu G.; Du Y.; Guo Y.; Meng Y.; Gai Z.; Zhang M.; Wang J.; deMello A. Plasmofluidic-Based Near-Field Optical Trapping of Dielectric Nano-Objects Using Gold Nanoislands Sensor Chips. ACS Appl. Mater. Interfaces 2022, 14, 47409–47419. 10.1021/acsami.2c12651. 36240070

[ref8] Bhalla N.; Sathish S.; Sinha A.; Shen A. Q. Large-Scale Nanophotonic Structures for Long-Term Monitoring of Cell Proliferation. Advanced Biosystems 2018, 2, 1700258 10.1002/adbi.201870031.

[ref9] Funari R.; Bhalla N.; Chu K.-Y.; Soderstrom B.; Shen A. Q. Nanoplasmonics for real-time and label-free monitoring of microbial biofilm formation. ACS Sens. 2018, 3, 1499–1509. 10.1021/acssensors.8b00287. 30062880

[ref10] Bhalla N.; Sathish S.; Galvin C. J.; Campbell R. A.; Sinha A.; Shen A. Q. Plasma-assisted large-scale nanoassembly of metal-insulator bioplasmonic mushrooms. ACS Appl. Mater. Interfaces 2018, 10, 219–226. 10.1021/acsami.7b15396. 29236477

[ref11] Roether J.; Chu K.-Y.; Willenbacher N.; Shen A. Q.; Bhalla N. Real-time monitoring of DNA immobilization and detection of DNA polymerase activity by a microfluidic nanoplasmonic platform. Biosens. Bioelectron. 2019, 142, 111528 10.1016/j.bios.2019.111528. 31362202

[ref12] Nguyen A. H.; Lee J.; Choi H. I.; Kwak H. S.; Sim S. J. Fabrication of plasmon length-based surface enhanced Raman scattering for multiplex detection on microfluidic device. Biosens. Bioelectron. 2015, 70, 358–365. 10.1016/j.bios.2015.03.064. 25841120

[ref13] Haes A. J.; Van Duyne R. P. A nanoscale optical biosensor: sensitivity and selectivity of an approach based on the localized surface plasmon resonance spectroscopy of triangular silver nanoparticles. J. Am. Chem. Soc. 2002, 124, 10596–10604. 10.1021/ja020393x. 12197762

[ref14] Duan Q.; Liu Y.; Chang S.; Chen H.; Chen J. Surface plasmonic sensors: Sensing mechanism and recent applications. Sensors 2021, 21, 5262 10.3390/s21165262. 34450704 PMC8401600

[ref15] Gong M.; Alamri M.; Ewing D.; Sadeghi S. M.; Wu J. Z. Localized surface plasmon resonance enhanced light absorption in AuCu/CsPbCl3 core/shell nanocrystals. Adv. Mater. 2020, 32, 2002163 10.1002/adma.202002163. 32449564

[ref16] Waitkus J.; Chang Y.; Liu L.; Puttaswamy S. V.; Chung T.; Vargas A. M. M.; Dollery S. J.; O’Connell M. R.; Cai H.; Tobin G. J. Gold Nanoparticle Enabled Localized Surface Plasmon Resonance on Unique Gold Nanomushroom Structures for On-Chip CRISPR-Cas13a Sensing. Advanced Materials Interfaces 2023, 10, 2201261 10.1002/admi.202370004. 37091050 PMC10121183

[ref17] Yao Y.; Hu T.; Chai Y.; Ju J.; Zhang J.; Shen W.; Shi H.; Liu C.; Huang C.; Tang S. A rapid “cusp-covering” to Au nanostar as plasmonic sensor in a single-drop microreactor for the determination of kanamycin in biosamples. Sens. Actuators, B 2022, 366, 131993 10.1016/j.snb.2022.131993.

[ref18] Jeon H. B.; Tsalu P. V.; Ha J. W. Shape effect on the refractive index sensitivity at localized surface plasmon resonance inflection points of single gold nanocubes with vertices. Sci. Rep. 2019, 9, 13635 10.1038/s41598-019-50032-3. 31541135 PMC6754453

[ref19] Kaja S.; Mathews A. V.; Venuganti V. V. K.; Nag A. Bimetallic Ag-Cu Alloy SERS Substrates as Label-Free Biomedical Sensors: Femtomolar Detection of Anticancer Drug Mitoxantrone with Multiplexing. Langmuir 2023, 39, 5591–5601. 10.1021/acs.langmuir.3c00525. 37025057

[ref20] Rahmani H.; Mahjoub A. R.; Khazaee Z. Bimetallic CuAg Alloyed Nanoparticles Anchored on CdS Nanorods for the Photocatalytic Degradation of Enrofloxacin. ACS Appl. Nano Mater. 2023, 6, 4554–4566. 10.1021/acsanm.3c00039.

[ref21] Mandal N.; Das A.; Moirangthem R. S. In-situ label-free optical biosensing with plasmonic enhanced ellipsometry using partially-embedded bimetallic Ag-Au alloy nanoparticles. Sens. Actuators, B 2023, 379, 133164 10.1016/j.snb.2022.133164.

[ref22] Liu L.; Zhang H.; Xing S.; Zhang Y.; Shangguan L.; Wei C.; Peng F.; Liu X. Copper-Zinc Bimetallic Single-Atom Catalysts with Localized Surface Plasmon Resonance-Enhanced Photothermal Effect and Catalytic Activity for Melanoma Treatment and Wound-Healing. Advanced Science 2023, 10, 2207342 10.1002/advs.202207342. 37096842 PMC10288238

[ref23] Liao Z.; Peng X.; Liu L.; Xu Y.; Xu K.-D.; Pan B.; Luo G. Q.; Liu Y. Microwave Plasmonic Exceptional Points for Enhanced Sensing. Laser and Photonics Reviews 2023, 17, 2300276 10.1002/lpor.202300276.

[ref24] Li G.; Singh R.; Guo J.; Zhang B.; Kumar S. Nb2CTx MXene-assisted double S-tapered fiber-based LSPR sensor with improved features for tyramine detection. Appl. Phys. Lett. 2023, 122, 083701 10.1063/5.0143776.

[ref25] Bhalla N.; Payam A. F.; Morelli A.; Sharma P. K.; Johnson R.; Thomson A.; Jolly P.; Canfarotta F. Nanoplasmonic biosensor for rapid detection of multiple viral variants in human serum. Sens. Actuators, B 2022, 365, 131906 10.1016/j.snb.2022.131906. PMC9015716 35463481

[ref26] Bhalla N.; Jamshaid A.; Leung M. H.; Ishizu N.; Shen A. Q. Electrical contact of metals at the nanoscale overcomes the oxidative susceptibility of silver-based nanobiosensors. ACS Appl. Nano Mater. 2019, 2, 2064–2075. 10.1021/acsanm.9b00066.

[ref27] Bhalla N.; Lee D.; Sathish S.; Shen A. Q. Dual-mode refractive index and charge sensing to investigate complex surface chemistry on nanostructures. Nanoscale 2017, 9, 547–554. 10.1039/C6NR07664E. 27892593

[ref28] Murphy C. J.; Gole A. M.; Stone J. W.; Sisco P. N.; Alkilany A. M.; Goldsmith E. C.; Baxter S. C. Gold nanoparticles in biology: beyond toxicity to cellular imaging. Acc. Chem. Res. 2008, 41, 1721–1730. 10.1021/ar800035u. 18712884

[ref29] Menichetti A.; Mavridi-Printezi A.; Mordini D.; Montalti M. Effect of Size, Shape and Surface Functionalization on the Antibacterial Activity of Silver Nanoparticles. Journal of Functional Biomaterials 2023, 14, 244 10.3390/jfb14050244. 37233354 PMC10219039

[ref30] Wu Y.-L.; Putcha N.; Ng K. W.; Leong D. T.; Lim C. T.; Loo S. C. J.; Chen X. Biophysical responses upon the interaction of nanomaterials with cellular interfaces. Acc. Chem. Res. 2013, 46, 782–791. 10.1021/ar300046u. 23194178

[ref31] Li M.; Cushing S. K.; Zhang J.; Lankford J.; Aguilar Z. P.; Ma D.; Wu N. Shape-dependent surface-enhanced Raman scattering in gold-Raman-probe-silica sandwiched nanoparticles for biocompatible applications. Nanotechnology 2012, 23, 115501 10.1088/0957-4484/23/11/115501. 22383452

[ref32] Moghaddam R. K.; Bhalla N.; Shen A. Q.; Natale G. Deterministic particle assembly on nanophotonic chips. J. Colloid Interface Sci. 2021, 603, 259–269. 10.1016/j.jcis.2021.06.120. 34214719

[ref33] Miranda B.; Chu K.-Y.; Maffettone P. L.; Shen A. Q.; Funari R. Metal-enhanced fluorescence immunosensor based on plasmonic arrays of gold nanoislands on an etched glass substrate. ACS Appl. Nano Mater. 2020, 3, 10470–10478. 10.1021/acsanm.0c02388.

[ref34] Bhalla N.; Lee D.; Sathish S.; Shen A. Q. Dual-mode refractive index and charge sensing to investigate complex surface chemistry on nanostructures. Nanoscale 2017, 9, 547–554. 10.1039/C6NR07664E. 27892593

[ref35] Bhalla N.; Chiang H.-J.; Shen A. Q. Methods in Cell Biology; Elsevier: 2018; Vol. 148, pp 203–227. 30473070 10.1016/bs.mcb.2018.09.009

[ref36] Bhalla N.; Jain A.; Lee Y.; Shen A. Q.; Lee D. Dewetting metal nanofilms—Effect of substrate on refractive index sensitivity of nanoplasmonic gold. Nanomaterials 2019, 9, 1530 10.3390/nano9111530. 31717894 PMC6915419

[ref37] Krzywiecki M.; Grządziel L.; Bodzenta J.; Szuber J. Comparative study of surface morphology of copper phthalocyanine ultra thin films deposited on Si (111) native and RCA-cleaned substrates. Thin Solid Films 2012, 520, 3965–3970. 10.1016/j.tsf.2012.01.018.

[ref38] Bhalla N.; Di Lorenzo M.; Pula G.; Estrela P. Protein phosphorylation analysis based on proton release detection: Potential tools for drug discovery. Biosens. Bioelectron. 2014, 54, 109–114. 10.1016/j.bios.2013.10.037. 24252767

[ref39] Kern W. The evolution of silicon wafer cleaning technology. J. Electrochem. Soc. 1990, 137, 1887 10.1149/1.2086825.

[ref40] Park J.-G.; Lee S.-H.; Ryu J.-S.; Hong Y.-K.; Kim T.-G.; Busnaina A. A. Interfacial and electrokinetic characterization of IPA solutions related to semiconductor wafer drying and cleaning. J. Electrochem. Soc. 2006, 153, G811–G814. 10.1149/1.2214532.

[ref41] Tesler A. B.; Maoz B. M.; Feldman Y.; Vaskevich A.; Rubinstein I. Solid-state thermal dewetting of just-percolated gold films evaporated on glass: development of the morphology and optical properties. J. Phys. Chem. C 2013, 117, 11337–11346. 10.1021/jp400895z.

[ref42] Mayer-Hamblett N.; Aitken M. L.; Accurso F. J.; Kronmal R. A.; Konstan M. W.; Burns J. L.; Sagel S. D.; Ramsey B. W. Association between pulmonary function and sputum biomarkers in cystic fibrosis. American Journal of Respiratory and Critical Care Medicine 2007, 175, 822–828. 10.1164/rccm.200609-1354OC. 17234902 PMC2720115

[ref43] Kuiper J. R.; O’Brien K. M.; Ferguson K. K.; Buckley J. P. Urinary specific gravity measures in the US population: Implications for the adjustment of non-persistent chemical urinary biomarker data. Environ. Int. 2021, 156, 106656 10.1016/j.envint.2021.106656. 34062395 PMC8380693

[ref44] Shaikh N.; Shope M. F.; Kurs-Lasky M. Urine specific gravity and the accuracy of urinalysis. Pediatrics 2019, 144, e20190467 10.1542/peds.2019-0467. 31578222

[ref45] Oppliger R. A.; Magnes S. A.; Popowski L. A.; Gisolfi C. V. Accuracy of urine specific gravity and osmolality as indicators of hydration status. International Journal of Sport Nutrition and Exercise Metabolism 2005, 15, 236–251. 10.1123/ijsnem.15.3.236. 16131695

[ref46] Berry E. M.; Gupta M. M.; Turner S. J.; Burns R. R. Variation in plasma calcium with induced changes in plasma specific gravity, total protein, and albumin. Br Med. J. 1973, 4, 640–643. 10.1136/bmj.4.5893.640. 4758543 PMC1587667

[ref47] Bhalla N. Human Urine Specific Gravity Detection using Nanoplasmonics: A Paradigm Shift from Scales to Biosensors. Advanced Sensor Research 2024, 3, 2300115 10.1002/adsr.202300115.

[ref48] Mao W.; Zhang H.; Xu Z.; Geng J.; Zhang Z.; Wu J.; Xu B.; Chen M. Relationship between urine specific gravity and the prevalence rate of kidney stone. Translational Andrology and Urology 2021, 10, 184 10.21037/tau-20-929. 33532308 PMC7844516

[ref49] Tang J.; Qiu G.; Zhang X.; Wang J. A 3D-cascade-microlens optofluidic chip for refractometry with adjustable sensitivity. Lab Chip 2021, 21, 3784–3792. 10.1039/D1LC00570G. 34581391

[ref50] Grzybowski B. A.; Wilmer C. E.; Kim J.; Browne K. P.; Bishop K. J. Self-assembly: from crystals to cells. Soft Matter 2009, 5, 1110–1128. 10.1039/b819321p.

[ref51] Jiang S.; Van Dyk A.; Maurice A.; Bohling J.; Fasano D.; Brownell S. Design colloidal particle morphology and self-assembly for coating applications. Chem. Soc. Rev. 2017, 46, 3792–3807. 10.1039/C6CS00807K. 28470250

[ref52] Zhang Y.; Chen Y.; Nie Y.; Yang Z.; Yuan R.; Wang H.; Chai Y. Highly Efficient Aggregation-Induced Electrochemiluminescence of Al (III)-Cbatpy Metal-Organic Gels Obtained by Ultrarapid Self-Assembly for a Biosensing Application. Anal. Chem. 2022, 94, 12196–12203. 10.1021/acs.analchem.2c02669. 35996222

[ref53] Fritschen A.; Blaeser A. Biosynthetic, biomimetic, and self-assembled vascularized Organ-on-a-Chip systems. Biomaterials 2021, 268, 120556 10.1016/j.biomaterials.2020.120556. 33310539

[ref54] Tang X.; Liu X.; Li P.; Liu F.; Kojima M.; Huang Q.; Arai T. On-Chip cell-cell interaction monitoring at single-cell level by efficient immobilization of multiple cells in adjustable quantities. Anal. Chem. 2020, 92, 11607–11616. 10.1021/acs.analchem.0c01148. 32605365

[ref55] Anggraini D.; Ota N.; Shen Y.; Tang T.; Tanaka Y.; Hosokawa Y.; Li M.; Yalikun Y. Recent advances in microfluidic devices for single-cell cultivation: methods and applications. Lab Chip 2022, 22, 1438–1468. 10.1039/D1LC01030A. 35274649

[ref56] Piradashvili K.; Alexandrino E. M.; Wurm F. R.; Landfester K. Reactions and polymerizations at the liquid-liquid interface. Chem. Rev. 2016, 116, 2141–2169. 10.1021/acs.chemrev.5b00567. 26708780

[ref57] Liang L.; Wen T.; Xin J.; Su C.; Song K.; Zhao W.; Liu H.; Su G. Fluoropolymer: A Review on Its Emulsion Preparation and Wettability to Solid-Liquid Interface. Molecules 2023, 28, 905 10.3390/molecules28020905. 36677962 PMC9866989

[ref58] Yang L.; Feng J.; Wang J.-N.; Gao Z.; Xu J.; Mei Y.; Song Y.-Y. Engineering large-scaled electrochromic semiconductor films as reproductive SERS substrates for operando investigation at the solid/liquid interfaces. Chin. Chem. Lett. 2022, 33, 5169–5173. 10.1016/j.cclet.2022.03.011.

[ref59] Sendner C.; Horinek D.; Bocquet L.; Netz R. R. Interfacial water at hydrophobic and hydrophilic surfaces: Slip, viscosity, and diffusion. Langmuir 2009, 25, 10768–10781. 10.1021/la901314b. 19591481

[ref60] Ho T. A.; Papavassiliou D. V.; Lee L. L.; Striolo A. Liquid water can slip on a hydrophilic surface. Proceedings of the National Academy of Sciences 2011, 108, 16170–16175. 10.1073/pnas.1105189108. PMC3182716 21911406

[ref61] Yagita T.; Ito T.; Hirano T.; Toyomasu T.; Hasegawa S.; Saito T.; Fujisawa S. Evaluating the Emulsifying Capacity of Cellulose Nanofibers Using Inverse Gas Chromatography. Langmuir 2023, 39, 4362–4369. 10.1021/acs.langmuir.2c03369. 36917026

[ref62] Fukuyama M.; Kubota K.; Hibara A. Nanoparticle Assembly at the Water-Oil Interface Induced by Spontaneous Emulsification for Microdroplet Immunoassay. Langmuir 2023, 39, 7884–7890. 10.1021/acs.langmuir.3c00723. 37218677

[ref63] Zhang H.; Zhang Z.; Grauby-Heywang C.; Kellay H.; Maali A. Air/Water Interface Rheology Probed by Thermal Capillary Waves. Langmuir 2023, 39, 3332–3340. 10.1021/acs.langmuir.2c03193. 36802344

[ref64] Olszewski M.; Hu X.; Lin T.-C.; Matyjaszewski K.; Lebedeva N.; Taylor P. Oscillatory and Relaxation Study of the Interfacial Rheology of Star Polymers with Low-Grafting-Density PEO Arms and Hydrophobic Poly (divinylbenzene) Cores. Langmuir 2023, 39, 7741–7758. 10.1021/acs.langmuir.3c00557. 37216597

[ref65] Shen A. Q.; Gleason B.; McKinley G. H.; Stone H. A. Fiber coating with surfactant solutions. Phys. Fluids 2002, 14, 4055–4068. 10.1063/1.1512287.

[ref66] Chen E.; Browne C.; Haward S.; Shen A.; Datta S. Influence of geometric ordering on viscoelastic flow instabilities in 3D porous media. APS March Meeting, 2023.

[ref67] Datta S. S.; Ardekani A. M.; Arratia P. E.; Beris A. N.; Bischofberger I.; McKinley G. H.; Eggers J. G.; López-Aguilar J. E.; Fielding S. M.; Frishman A. Perspectives on viscoelastic flow instabilities and elastic turbulence. Physical Review Fluids 2022, 7, 080701 10.1103/PhysRevFluids.7.080001.

[ref68] Langevin D. Rheology of adsorbed surfactant monolayers at fluid surfaces. Annu. Rev. Fluid Mech. 2014, 46, 47–65. 10.1146/annurev-fluid-010313-141403.

[ref69] Funari R.; Shen A. Q. Detection and characterization of bacterial biofilms and biofilm-based sensors. ACS Sens. 2022, 7, 347–357. 10.1021/acssensors.1c02722. 35171575

